# A Novel In Vitro Scrubber Model for Evaluating Wound Cleansing on Biofilms

**DOI:** 10.1111/wrr.70063

**Published:** 2025-07-31

**Authors:** Fergus Watson, Marcus J. Swann, Jeanne Saint Bezard, Rui Chen, Alisha Oropallo, Steven L. Percival

**Affiliations:** ^1^ 5D Health Protection Group Limited Liverpool UK; ^2^ Comprehensive Wound Healing Center and Hyperbarics, Northwell Health New York New York USA

**Keywords:** antimicrobial, biofilm, cleansing, hygiene, wound

## Abstract

Chronic wounds are a significant burden on patients and hospitals globally, with all exhibiting high microbial loading and biofilm. Wound cleansing is critical for removing foreign contaminants, damaged tissue and opportunistic pathogens, allowing for improved healing. This study addresses the need for a standardised in vitro preclinical model for comparing the efficacy of antimicrobial wound cleansing solutions with mechanical disruption on biofilms. A novel model was developed to emulate cleaning practises whilst standardising pressure applied and scrubbing forces applied to the wound bed. Using wounded porcine explants, microbial biofilms were formed on the surface before exposure to varying physical parameters and cleaning solutions. The results showed that both increased pressure and scrubbing duration had a positive impact on biofilm removal, demonstrating > 1 log reduction in microbial levels. The model was able to show the significant difference in cleaning solutions between saline and an antimicrobial‐based solution (HClO), > 4.5 log reduction, whilst under identical conditions. Confocal laser microscopy, using fluorescent viability stains, provided supporting evidence of biofilm disruption using gauze. The model's adaptability and versatility help to provide clinically relevant in vitro evidence and effective comparisons of wound cleansing agents on biofilms through the standardisation of different cleaning techniques.

## 1 Introduction

Wound cleansing is a critical step of wound care, playing a pivotal role in preventing infections and promoting optimal healing conditions. The primary goal of wound cleansing is to remove debris, exudate, and contaminants from the wound bed, thereby reducing the microbial load and preventing the formation of biofilms [1, 2]. Biofilms are structured communities of microorganisms encased in a self‐produced extracellular matrix that often adhere to surfaces, including wound beds. These biofilms, and pathogenic microbes within, are notoriously difficult to eradicate due to the inherent protection and evasion they provide from the host's immune response and antimicrobial treatments [3]. The presence of biofilms in both acute and chronic wounds can lead to both quiet and chronic inflammation, delayed healing and increases the propensity of a systemic infection [4]. Therefore, effective wound cleansing is essential to prevent biofilm formation and disrupt the biofilm already present, creating an environment conducive to healing [5].

The process of wound cleansing involves the use of various solutions and techniques to irrigate and debride the wound. Saline and water are commonly used for their non‐cytotoxic properties, while other solutions, such as silver, iodine and polyhexanide, may be employed for their antimicrobial effects [6]. Scrubbing and irrigation are common methods used for wound cleansing, each with its own advantages and considerations regarding force and pressure. The choice of method and the amount of force or pressure applied during wound cleansing depends on several factors, including the wound type, location and the patient's overall condition [7]. Using the correct technique and applying the right amount of pressure are essential for promoting optimal wound healing and preventing secondary complications. In clinical practice, the choice of method and the application of force or pressure largely depend on the healthcare practitioner's experience, and this subjective approach results in a controversial area when comparing data and determining a positive outcome.

In addition to clinical practices, the significance of standardised in vitro testing in wound care cannot be overstated. This testing provides a consistent and reproducible method to evaluate the performance of wound care products under controlled conditions [8]. It enables researchers to compare different products and assess their effectiveness in preventing biofilm formation and promoting wound healing, correlating in vitro results to in vivo studies. Such testing is crucial for determining the efficacy and safety of wound care products, including cleansers, dressings and antimicrobial agents [9, 10] However, most in vitro evaluations are conducted without controlling physical parameters such as force and pressure, which are critical factors in effective clinical wound cleansing [11]. Currently, there is no standardised in vitro physical model that accurately mimics clinical wound scrubbing with controlled force, that is, vertical and horizontal forces. To address this gap, we have designed a novel in vitro physical wound scrubber model with adjustable forces and procedures for evaluating wound cleansing techniques. This model not only provides an advanced tool for assessing wound cleansing methods but also supports evidence‐based clinical practices and enhances patient care.

## 2 Methodology

### 2.1 Cleanser/Irrigation Solutions

To assess wound cleansing practice within this model, three commonly used wound irrigating/cleanser solutions were employed [12, 13]; a saline (0.9% sodium chloride) wash, a poloxamer surfactant‐based solution (20%) produced in‐house using poloxamer 188, and a commerically‐available wound cleansing solution containing hypochlorous acid as an antimicrobial preservative ingredient, referred to herein as antimicrobial‐based solution (≤ 0.033%) (Vashe, Urgo).

### 2.2 Bacterial Cultures

In this study, *Pseudomonas aeruginosa* ATCC 15442 was used for generating the biofilm model; stock cultures of the strain were obtained from cryovial stocks and cultured in tryptone soya broth (TSB) overnight at 37°C. The following day, the overnight culture was resuspended to 0.5 McFarland (1 × 10^8^ colony forming unit [CFU/mL]) before being adjusted to a concentration of approximately 10^6^ CFU/mL in TSB.

### 2.3 Porcine Skin Biofilm Model

Porcine skin explants were obtained from a local butcher (Local butcher, Lymm, UK), as a by‐product of the meat industry, and processed or frozen within 72 h of slaughter. All fat was removed from the underside of the explant skin, which was then were cut into 4 × 4 cm sections before using a 12 mm (diameter) biopsy punch to ‘wound’ the surface by removing approximately 1.0–2.0 mm of the upper layers of skin, including the epidermis, to reveal the dermis beneath and simulate a wound bed. Once cut, the samples were sterilised in 4% sodium hypochlorite for 4 h and then washed three times with sterile phosphate‐buffered saline (PBS) and stored in sterile PBS 24 h prior to use [14].

The simulated wound bed of the porcine skin was then inoculated with 50 μL of the adjusted overnight culture and placed into a sterile jar and incubated at 37°C for 24 h. Following incubation, the skin samples were gently washed to remove any planktonic and loosely attached cells before being mounted, with a cyanoacrylate adhesive, to the base of a sterile Petri dish and transferred to the wound cleansing model. At this stage, an untreated control sample was removed and processed, as per biofilm analysis stages below, to determine the viability of the biofilms in the model.

### 2.4 Wound Cleansing Model

**FIGURE 1:**
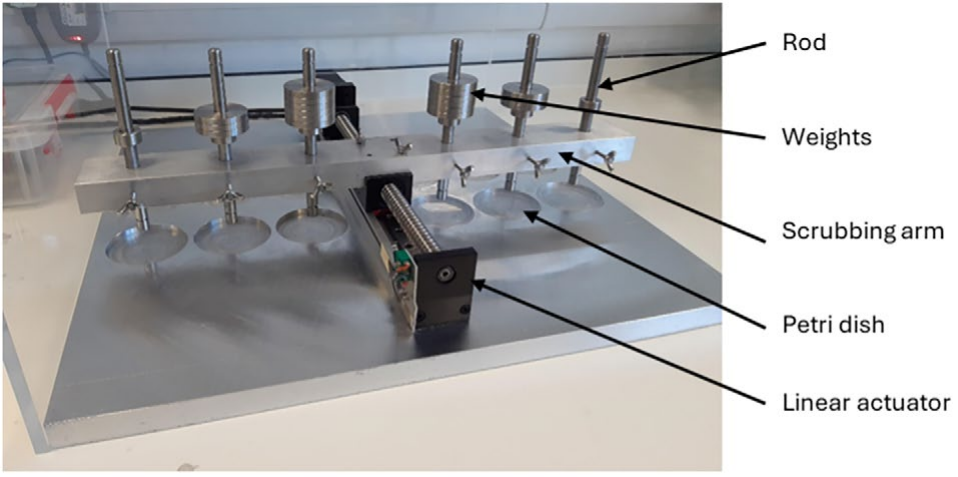
Wound cleansing model.

A novel wound cleansing model was developed to emulate the physical interactions experienced by patients during wound cleansing, such as cleaning and scrubbing of cleanser‐soaked gauze that are used routinely in clinical practice (Figure 1). The model consisted of a linear actuator to allow horizontal movement and a weighted rod which was free to move vertically, onto which a Multisorb gauze material (BSN medical, GmBH, Germany) was attached to apply vertical pressure on the sample (Design available upon reasonable request). Weights were added to the rod to vary the pressure exerted over the 8 mm diameter contact area of the wound. The applied pressure ranged between < 2.6 PSI (182 g/cm^2^) for the unweighted rod to 14.6 PSI for the maximum applied weight used in this study (1026 g/cm^2^). Prior to use, the gauze mounted rods were lowered into the respective cleanser solutions and left to absorb for up to 5 min. The rods were then raised, and any excess solution was allowed to drain off for 30 s, resulting in approximately 2.8 ± 0.2 g of cleanser solution absorbed into each gauze.

The cleanser‐soaked gauzes were then placed centrally on top of the wounded porcine skin samples and subjected to variable protocols to assess the impacts of pressure, scrubbing duration and solution types on the removal and inactivation of porcine biofilms. The scrubbing process involved moving the rod‐mounted gauze backwards and forwards across the simulated wound; effective scrubbing speed was 1.6 cm/s and had a total distance of 3.4 cm.

A breakdown of these protocol variants can be seen within Table 1 and is based upon standard clinical practice of a 10‐min soak followed by 30 s of scrubbing [6]. These include: Protocol 1 which assessed biofilm growth within the model (untreated control); Protocol 2 which assessed the impact of soaking in the absence of weight and scrubbing; Protocol 3–5 which assessed the impact of pressure (minimum: 3 PSI/maximum: 15 PSI); Protocol 6–8 which assessed the impact of scrubbing (minimum: 0 s/maximum: 60 s); and Protocol 9–11 which assessed the impact of the cleansing solution (saline/surfactant/antimicrobial).

**TABLE 1:** Details of the protocols used to assess pressure, scrubbing duration and cleansing solution; and the subsequent log reduction achieved in comparison with the untreated control.

Protocol no.	Variant	Pressure applied (PSI)	Soak duration (s)	Scrubbing duration (s)	Wound cleansing solution	Log reduction
1	Untreated control	0	0	0	None	—
2^a^	Soaking only	0	600	0	Saline	0.37
3	Pressure applied	3	600	30	Saline	1.51
4^b^		9	600	30	Saline	1.78
5		15	600	30	Saline	1.65
6	Scrubbing duration	9	600	0	Saline	1.15
7^b^		9	600	30	Saline	1.78
8		9	600	60	Saline	2.07
9^b^	Cleansing solution	9	600	30	Saline	1.78
10		9	600	30	Surfactant	1.74
11		9	600	30	Antimicrobial	> 4.76

^a^ Weight of dressing without additional weights applied was < 2.6 PSI.

^b^ Data sets for protocols 4, 7 and 9 are identical.

### 2.5 Biofilm Analysis: Recovery

After each protocol shown above was performed, the biofilm samples were removed using an 8 mm (diameter) biopsy punch. A section of the centre of the wound bed was removed and transferred into neutralising broth. The samples were then sonicated on full power for 30 min, before being briefly vortexed and aliquoted for enumeration. The aliquots were serially diluted and plated on tryptone soya agar (TSA) to determine the total viability of microbial bioburden remaining.

### 2.6 Statistical Analysis

Each experimental protocol included three biological replicates. The number of CFU per plate was enumerated and raw data input into Microsoft Excel to calculate the microbial loading using the following formula:

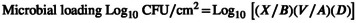




where *X* is the average CFU, *B* the volume plated, *V* the final volume, *A* the surface area, *D* the dilution.

The log reduction of each test variable was calculated by subtracting the average log value of each treatment from the average log value of the untreated negative control. A limit of detection of 2.3 log was used in this study. To determine if there was a statistical difference in the average microbial loading values between the treated and untreated biofilms, a Student's *t*‐test was performed using Microsoft Excel.

### 2.7 Biofilm Analysis: Microscopy

Samples of the 8 mm sections were subjected to confocal laser scanning microscopy analysis. The resultant biofilms were stained with LIVE/DEAD Baclight fluorescent stains adjusted to a final concentration of 2.5 μM of SYTO 9 and 27.5 μM of propidium iodide before being incubated at room temperature in the dark for 20 min. The stain was then aspirated, and the biofilms were washed carefully with PBS; 20 μL of PBS was subsequently added to each well to ensure the biofilm remained hydrated. The stained biofilms were visualised with an LSM 780 Zeiss confocal microscope with a ×20 (0.9 NA) air objective. A porcine skin sample (*n* = 1) from each protocol was imaged across three fields of view, and all images were taken under identical conditions. The images were analysed using Fiji ImageJ software.

## 3 Results

**FIGURE 2:**
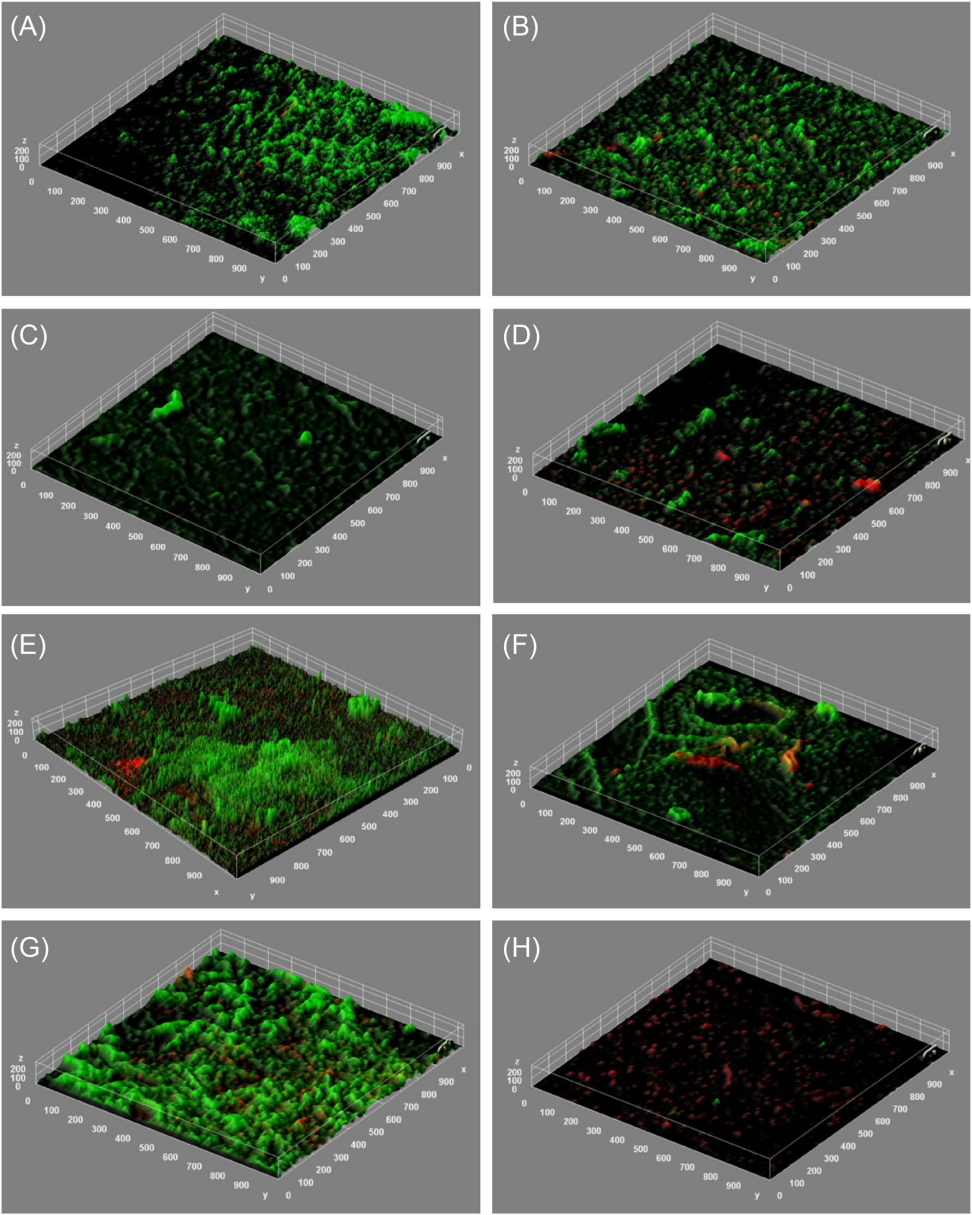
Representative micrographs of the biofilm model samples, using *P. aeruginosa* stained with viability stains to highlight viable (green) and non‐viable (red) populations of bacterial cells. These include: (A) Protocol 1, for the untreated control; (B) Protocol 2, for the soak‐only control; (C) Protocol 3, for minimum pressure applied; (D) Protocol 5, for maximum pressure applied; (E) Protocol 6 for minimum scrubbing duration; (F) Protocol 8, for maximum scrubbing duration; (G) Protocol 9, for saline wound cleanser; (H) Protocol 11, for antimicrobial‐based wound cleanser.

The porcine skin model produced confluent biofilms on the surface of the wound with microbial loading of (mean) 7.06 ± 0.09 Log_10_ CFU/cm^2^, as shown within Figure 2A. These models were then subjected to cleansing with variances in pressure and force using an increasing number of weights, in scrubbing duration and in cleansing solutions, for example, saline, surfactant‐based and antimicrobial‐based solution. For comparison, a sample of the porcine skin models were subjected to soaking without scrubbing in order to distinguish the impact of shear within the cleansing model. Following a 10‐min soak with saline, achieved by applying the cleanser‐soaked gauze to the surface of the biofilm with < 2.6 PSI pressure, only a 0.37 log reduction was observed (*p* = 0.03). This was supported by no identifiable change in viable cells (green) on the surface of the porcine wound bed during microscopy (Figure 2B).

### 3.1 Pressure

**FIGURE 3:**
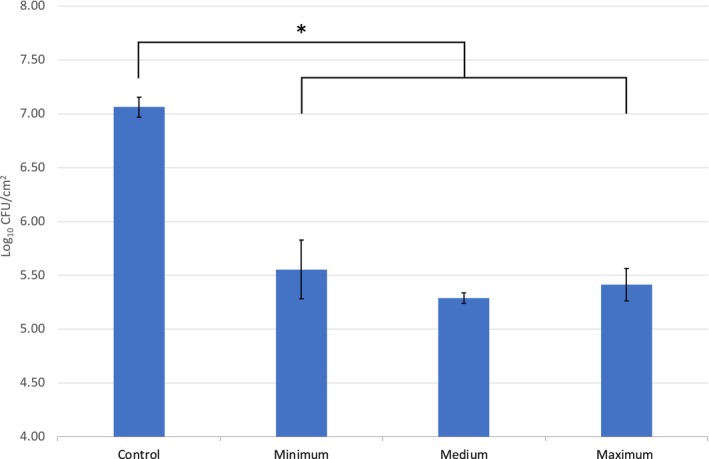
Microbial loadings data (Log_10_ CFU/cm^2^) to show the impact of pressure on biofilm reduction on the porcine skin samples. The pressure range included 3 PSI (minimum), 9 PSI (medium) and 15 PSI (maximum) applied for a duration of 30 s post 600 s of soaking within saline solution. A significant reduction in bioburden was identified in comparison with the untreated negative control as indicated, *p < 0.01.

To show the impact of the pressure used during wound cleansing, pressures varying between 3 and 15 PSI were applied to samples of the porcine skin models during soaking (600 s) and scrubbing (30 s). Whilst the largest average log reduction with the lowest standard deviation was observed with medium pressure, and the smallest log reduction with the largest standard deviation was observed for the samples with the lowest applied pressure, no significant direct correlation between weight in the 3–15 PSI range and removal of microbial contamination could be shown (Figure 3). Nonetheless, a significant log reduction (average: 1.64 ± 0.13) across all three variants was observed in comparison to the untreated (negative control) wound samples (*p* < 0.01), as seen within the biofilm images (Figure 2C,D); additionally, a log reduction of 1.27 in comparison to the unscrubbed *soak only* wound samples was found.

### 3.2 Scrubbing Duration

**FIGURE 4:**
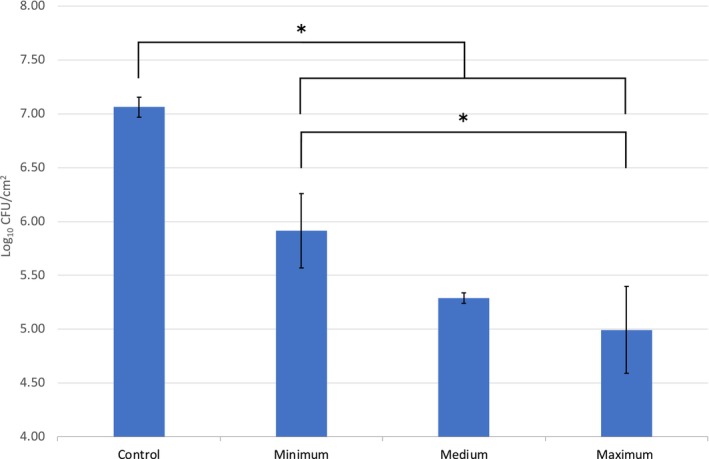
Microbial loadings data (Log_10_ CFU/cm^2^) to show the impact of scrubbing duration (s) on biofilm reduction on the porcine skin samples. The scrubbing durations range included 0 s (minimum), 30 s (medium) and 60 s (maximum) applied using 9 PSI of pressure and 600 s of soaking within saline solution. A significant reduction in bioburden was identified in comparison with the untreated negative control as indicated; additionally, a significant difference was observed between the minimum and maximum variables, *p < 0.01.

The duration for scrubbing during wound cleansing tested ranged from 0 to 60 s with a constant pressure of 9 PSI. Here, the data did demonstrate a correlation with the length of scrub and a reduction in microbial concentration within the biofilms, shown by the log reduction increasing from 1.15 at 0 s to 2.07 after 60 s (Figure 4). All of the scrubbing durations tested had significantly lower microbial counts than those obtained from untreated controls, as well as the unscrubbed *soak only* samples (*p* < 0.01). This was supported by the confocal laser microscope micrographs which indicated a reduction in the total population of viable cells (green) within the biofilm and an abundance of non‐viable cells (red) (Figure 2E,F).

### 3.3 Wound Cleansing Solutions

**FIGURE 5:**
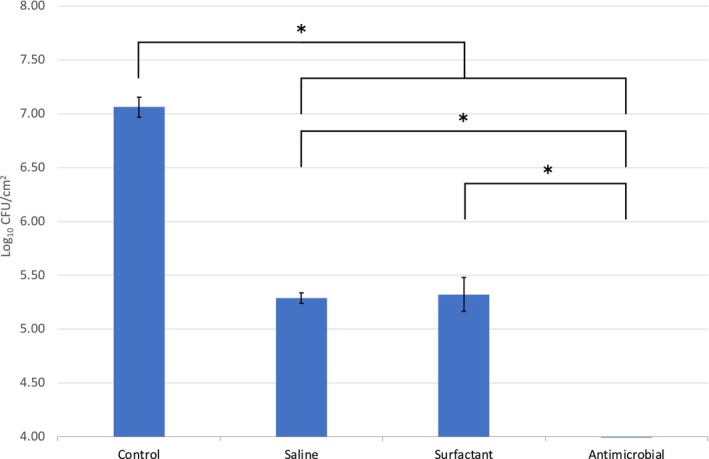
Microbial loadings data (Log_10_ CFU/cm^2^) to show the impact of cleanser solution on biofilm reduction on the porcine skin samples. The cleanser solutions included saline, a surfactant‐based solution, and an antimicrobial‐based solution, applied using 9 PSI of pressure with 600 s of soaking. A significant reduction in bioburden was identified in comparison with the untreated negative control as indicated; additionally, a significant difference was observed between the antimicrobial‐based solution and the saline and surfactant‐based solutions, *p < 0.01.

Common wound cleansing solutions often utilise a surfactant agent or an antimicrobial preservative within the formulation to enhance performance. Nonetheless, saline still remains a frequently used alternative in part due to its accessibility and lower costs [15]; within this study, all three variants were compared using a routine clinically relevant protocol (10‐min soak followed by 30 s of scrubbing with medium applied pressure). As shown, all three irrigating/cleansing solutions demonstrated an impact on the biofilm demonstrating a significant decrease in viability when compared to the controls (*p* < 0.01) (Figure 5). However, no significant difference was shown between the saline and the surfactant‐based cleanser, achieving only a 1.78 and 1.74 log reduction, respectively (*p* = 0.73). In contrast, complete inactivation of the microbes in the biofilm, and biofilm reduction through imaging, was shown for the antimicrobial‐based cleanser equivalent to greater than a 4.50 log reduction (Figure 2G,H). This was significantly lower in microbial counts than was achieved using the saline or surfactant‐based cleanser (*p* < 0.01).

## 4 Discussion

Cleansing is considered one of the most important stages necessary in wound management and wound bed preparation; and is very important in the removal of dead tissue, wound exudate, slough, foreign bodies, microorganisms, biofilms and metabolic waste [9]. Scrubbing is considered a form of mechanical debridement often used before dressing changes or other therapeutic practices [15]. It is internationally recognised and discussed amongst clinicians that insufficient wound cleansing is a key driver in biofilm formation and/or persistence resulting in chronic infections and associated morbidities depending on the proximity of the wound on the patient [16]. As stated by a panel of global experts: ‘debridement is one of the most important treatment strategies against biofilms’ and ‘one of the critical principles of wound bed preparation’ [17]. There is conflicting information by manufacturers and clinicians regarding the recommended use of different techniques and cleansing solutions [18]; and the same panel, as mentioned, agree that in vitro methods with clinically relevant test conditions provide a useful screening tool for biofilm‐based wound and skin treatments [17]. In this study, using a standardised model, we successfully differentiated the effects of pressure, scrubbing duration and cleanser type on biofilm reduction in wounds, based on microbial numbers.

In most countries, scrubbing is the dominant practice, despite the risks of tissue damage and subsequent impairment to healing [19, 20]. Understandably, previous experience is a major factor in wound cleansing practice as opposed to the availability of evidence [7]; and it can change subjectively depending on the patient's morbidities, location of the wound and psychological state [21]. The latter of which is known to be more affected by scrubbing procedures compared with approaches which use irrigation [22]. As a result, it is difficult to determine the pressure applied during scrubbing [19], and therefore, for guidance within this study, the perpendicularly applied pressure values (i.e., PSI) emulate those employed during irrigation techniques as per clinical practice by healthcare professionals. Generally, a pressure, in PSI, for irrigation between 3 and 15 is considered an effective range for therapeutic wound cleansing whilst minimising the risks on patient safety and limiting damage to the wound bed and surrounding tissues, or periwound area and periwound microbiome [23]. However, it should be recognised that the lateral shear and frictional force obtained from the movement of the gauze across the wound bed surface is likely different from that experienced by the stream of irrigation used in alternative techniques. The data shown in this study indicate no notable advantage to increasing pressure significantly above 3 PSI for the protocols involving scrubbing; and whilst evidence suggests increasing pressure can be more effective at biofilm removal [24], this aligns with other research [25, 26, 27, 28]. The microscopy images demonstrate subjectively no changes in the concentration of viable cells within the sample post‐cleansing (Figure 2C,D). However, a potential increase in non‐viable cells within the biofilm was observed; these inactive cells can be commonly associated with basal layers of the biofilm matrix and may represent a population of non‐metabolically active microbes [29]. We postulate the disturbance caused by the physical act of debridement can remove the top layer of viable/active microbes, revealing the basal layers beneath which are often considered the root cause for biofilm recalcitrance to antimicrobial interventions in chronic infections and disease [30]. It should also be noted that the samples with medium pressure applied during the soak, in the absence of scrubbing (Protocol 6) did show increased log reduction over those for which there was minimal applied pressure during the soak (Protocol 2). This is presumably due to increased physical interaction between the gauze and the underlying biofilm, causing some disruption with applied pressure in the absence of scrubbing.

It is well recognised that removal of necrotic tissue, slough and biofilm is vital to improving wound healing; however, there is limited guidance on wound cleansing practices [31, 32]. There is limited evidence to support the duration for wound cleansing, and often this is defined by manufacturers as being associated with the inactivation or working time of the antimicrobial agents present within the cleanser solution whilst factoring for patient safety (i.e., cytotoxicity) [33, 34, 35]. In this instance, the protocol was based upon previously reported durations within the literature for treating chronic wound infections and showed a direct correlation with biofilm removal [6]. Clinicians may spend upwards of 10 min debriding wounds; Bahr et al. and Mustafi et al. both found gauze debridement similar to that employed in this study, and it can take (on average) 5 min to appropriately prepare the wound bed or clear the site of infection [36, 37, 38]. The extent of scrubbing is subjective and often a reflection of the experience and training of the clinician, where the individual is reliant on visual inspection of the wound bed to determine the required level of cleaning [39, 40, 41, 42]. The confocal laser microscope micrographs demonstrate a clear reduction in the biofilm matrix, as reported within the CFU values, and similarly show increased signs of non‐viable cells as a result of the biofilm disruption brought about by an increased scrubbing duration.

Numerous studies support the use of specific cleansing agents for their therapeutic advantages and antimicrobial properties [43, 44, 45, 46, 47]. However, in real‐world practice, the choice of solutions is dependent on the preference and experience of the clinician administering the treatment, the healthcare policy governing the patient and institution, what is available on the formulary, cost and product availability [6, 48]. In this instance, unsurprisingly, a significant decrease in the biofilm's microbial viability and presence on the surface of the wound bed was shown for the antimicrobial solution tested in comparison to the saline and surfactant‐based cleansers. Conversely, the surfactant‐only‐based cleanser failed to show any notable difference from the saline. A large proportion of readily available wound cleansers will incorporate surfactants or surface active agents which are known to reduce surface tensions between liquids and between liquid and solids to aid removal [2, 45]. Poloxamers are widely used in wound care and are categorised as a non‐ionic surfactant comprised of a central hydrophobic chain of polyoxypropylene adjacent to two hydrophilic chains of polyoxyethylene [49]. The antibiofilm properties of some surfactants have been well documented and are primarily responsible for modifying the adhesive properties of the microorganisms with the matrix of the biofilm; thus increasing the probability of removal and also helping to enhance the performance of an antimicrobial [2, 45, 50]. Whilst inconclusive, clinical evidence suggests non‐ionic surfactants do not enhance the removal of all types of soil contaminants within the wound when used at certain levels and without being combined with antimicrobials [51].

In contrast, the confocal laser microscope micrographs taken within this study for the hypochlorous acid wound cleanser demonstrate an enhanced removal of microbes and biofilms indicated by the reduced fluorescence signal for both Syto‐9 (viable cells) and propidium iodide (non‐viable cells). In this study, the antimicrobial agent employed comprised a hypochlorous acid, an oxidising antimicrobial preservative agent that can target proteins, amongst other components, within the extracellular polymeric matrix of the biofilm, potentially causing the structure to become destabilised [52]. Albeit not reported to reduce the biomass, the additional combined effect of scrubbing and hypochlorous acid significantly cleared the wound bed of the presence of biofilm [53]. Future work may focus on the reduction of biofilm‐related biomass, which would include both the viable and non‐viable microorganisms, as well as the extracellular polymeric substances of the biofilm matrix.

With regards to wound cleansing and scrubbing practices, there are no conclusive randomised clinical trials to support a common approach [54]; rather, there is literature making recommendations from the field rather than methods based on meaningful research and quantitative values such as pressure [7]. The design of this novel model enables a versatile approach to assessing several key variables, not just those tested here, including substrate materials, microbiological contamination and scrubbing clothes or cleanser‐soaked materials. A limitation of the design, to be addressed in future iterations, is linked to the repetitive motion of scrubbing back and forth during cleansing. In the late 1990s, Barr and Trevelyan reported that using circular motions which migrated away from the centre of the wound would help minimise the risks of recontamination and aid bacterial removal [55, 56].

Wound cleansing used for treating chronic wounds can be instrumental in removing the heavy growth levels of bacteria and biofilm matrix from the wound bed [57]. Chronicity in this situation is defined by a wound's failure to reach a timely reparative process within 3 months [58, 59]. Biofilms within this environment are considered mature and more difficult to treat, in part due to their structural complexity and integration with the host developed over an extended period [60]. Therefore, whilst the study presented here employs a representative substrate, porcine skin explant, we acknowledge the biofilm growth used is indicative only of an acute wound infection and a comparatively reduced microbial challenge than a mature and more developed biofilm such as those seen within chronic wounds [60]. The adaptability of the model allows for future studies to utilise biofilm growth exceeding 24 h and additionally other complexities within the biofilm matrix such as extracellular substances and slough. Additionally, the presence of blood flow, immune responses and multiple bacterial species are critical factors which may influence both the wound healing process and the removal in vivo. The model currently utilises a single‐species biofilm of *P. aeruginosa* chosen for its role in wound management and often considered the reason why chronic wounds do not heal; and whilst relevant this may not fully represent the complexity of polymicrobial biofilms in chronic wounds [61, 62]. The use of porcine skin as a substrate, while offering similarities to human skin, incurs differences in skin structure and composition that may influence biofilm formation and response to cleansing interventions. Despite these limitations, this in vitro scrubber model represents a significant advancement in wound cleansing research.

In conclusion, the findings from this study have direct implications for clinical practice, whereby clinicians can utilise this information to refine their wound cleansing techniques, ensuring sufficient scrubbing time to effectively disrupt and remove biofilms. This study demonstrates the capability of a novel in vitro biofilm wound cleansing model to emulate a variety of clinical practices with an equally variable number of outputs for end point analysis, under controlled laboratory conditions. Other end point analysis to be incorporated into the future for this model will be to include evaluating biomass removal such as the EPS present in biofilm matrices, known for its pro‐inflammatory properties; further comparative studies of other antimicrobial agents and wound cleansing solutions; incorporating various wound types and surfaces, such as burns and variable wound bed depth; and the use of multispecies biofilms for additional clinical relevance. By optimising wound cleansing practices, clinicians can improve biofilm control, reduce the risk of chronic infections and promote wound healing. The model can be used to evaluate new wound cleansing products and technologies, facilitating the development of more effective biofilm removal strategies.

Future iterations may look to address the impact of different gauze materials and roughness, which will impact the frictional forces applied, on biofilm removal efficacy, as well as different microbes commonly seen within wounds, such as Gram negative bacteria, fungal or yeast and anaerobic bacterial strains [63]. This could lead to the development of optimised gauze materials specifically designed for wound cleansing and biofilm disruption. Dental studies have suggested that passive ultrasonic irrigation (PUI), which utilises an oscillating file to mechanical debride in addition to an irrigation flush, may be superior for biofilm removal in comparison to alternative conventional irrigation techniques [64, 65]. An in vitro study by Eneide et al. demonstrated that the conventional needle irrigation technique was significantly less effective than other techniques, including passive sonic irrigation and PUI, the latter of which proved to be the most effective, as biofilm removal was perceived to be an advantage of the mechanical component [64]. However, there is a paucity of data on the combined effects of scrubbing and irrigation on biofilm removal, which may benefit from the incorporation of this model. Translation to animal or human models would be beneficial. It is also possible that the model can be adapted to study different biofilm‐forming species, providing insights into the species‐specific responses to various wound‐cleansing techniques.

## Conflicts of Interest

The authors Fergus Watson, Marcus J. Swann, Jeanne Saint Bezard, Rui Chen, and Steven L. Percival are employed by 5D Health Protection Group Ltd. The author Alisha Oropallo is a consultant to Urgo Medical, USA at the time of submission.

## Data Availability

The data that supports the findings of this study are available upon reasonable request from the corresponding author.
